# A hospital-site controlled intervention using audit and feedback to implement guidelines concerning inappropriate treatment of catheter-associated asymptomatic bacteriuria

**DOI:** 10.1186/1748-5908-6-41

**Published:** 2011-04-22

**Authors:** Barbara W Trautner, P Adam Kelly, Nancy Petersen, Sylvia Hysong, Harrison Kell, Kershena S Liao, Jan E Patterson, Aanand D Naik

**Affiliations:** 1 Houston Health Services Research and Development Center of Excellence, Michael E. DeBakey VA Medical Center, Houston, TX, USA; 2 Baylor College of Medicine, Houston, TX, USA; 3 Research Service, Southeast Louisiana Veterans Health Care System, New Orleans, LA, USA; 4 Tulane University School of Medicine, New Orleans, LA, USA; 5 Medicine Service, South Texas Veterans Healthcare System, San Antonio, TX, USA

## Abstract

**Background:**

Catheter-associated urinary tract infection (CAUTI) is one of the most common hospital-acquired infections. However, many cases treated as hospital-acquired CAUTI are actually asymptomatic bacteriuria (ABU). Evidence-based guidelines recommend that providers neither screen for nor treat ABU in most catheterized patients, but there is a significant gap between these guidelines and clinical practice. Our objectives are (1) to evaluate the effectiveness of an audit and feedback intervention for increasing guideline-concordant care concerning catheter-associated ABU and (2) to measure improvements in healthcare providers' knowledge of and attitudes toward the practice guidelines associated with the intervention.

**Methods/Design:**

The study uses a controlled pre/post design to test an intervention using audit and feedback of healthcare providers to improve their compliance with ABU guidelines. The intervention and the control sites are two VA hospitals. For objective 1 we will review medical records to measure the clinical outcomes of inappropriate screening for and treatment of catheter-associated ABU. For objective 2 we will survey providers' knowledge and attitudes. Three phases of our protocol are proposed: the first 12-month phase will involve observation of the baseline incidence of inappropriate screening for and treatment of ABU at both sites. This surveillance for clinical outcomes will continue at both sites throughout the study. Phase 2 consists of 12 months of individualized audit and feedback at the intervention site and guidelines distribution at both sites. The third phase, also over 12 months, will provide unit-level feedback at the intervention site to assess sustainability. Healthcare providers at the intervention site during phase 2 and at both sites during phase 3 will complete pre/post surveys of awareness and familiarity (knowledge), as well as of acceptance and outcome expectancy (attitudes) regarding the relevant practice guidelines.

**Discussion:**

Our proposal to bring clinical practice in line with published guidelines has significant potential to decrease overdiagnosis of CAUTI and associated inappropriate antibiotic use. Our study will also provide information about how to maximize effectiveness of audit and feedback to achieve guideline adherence in the inpatient setting.

**Trial Registration:**

NCT01052545

## Background

Urinary tract infection (UTI) is the single most common hospital-acquired infection, and many cases of nosocomial UTI are associated with an indwelling urinary catheter [1,2]. Urinary catheters bypass normal host defenses, and bacteriuria develops at the rate of approximately 5% per day; [3] nearly all individuals (98%) who are catheterized for 30 days or longer will have bacteriuria caused by one or more species of potentially pathogenic bacteria [4,5]. Furthermore, organisms causing catheter-associated urinary tract infection (CAUTI) are frequently resistant to one or more antibiotics [6,7]. In addition to these concerns regarding quality of care, annual incremental costs attributed to nosocomial CAUTI in 2002 were estimated to exceed $451 million [8]. Finally, since public reporting of nosocomial infections has become mandated in more than 30 states, [9] hospitals have strong incentive to reduce their rates of hospital-acquired CAUTI.

An important distinction exists between CAUTI and asymptomatic bacteriuria (ABU). CAUTI, as defined by the US Centers for Disease Control (CDC) and the National Healthcare Safety Network, involves symptoms (fever, urgency, frequency, dysuria, or suprapubic tenderness), in addition to microorganisms in the urine [10]. CAUTI requires antimicrobial treatment to relieve symptoms, while patients with ABU are, by definition, asymptomatic. Treatment of ABU in patients with an indwelling catheter does not improve morbidity or mortality, nor does it decrease the incidence of symptomatic CAUTI [11]. Use of antimicrobial agents to prevent or to treat catheter-associated ABU does lead to emergence of resistant flora, however [12]. Accordingly, evidence-based guidelines concerning ABU have stated that it is inappropriate to screen for or to treat ABU associated with the presence of a urinary catheter, with the exceptions of pregnant women and persons undergoing invasive urologic procedures [11,13]. The CDC campaign to prevent antimicrobial resistance in hospitalized patients likewise instructs clinicians to "treat infection, not colonization." [14] Unfortunately, a significant translation gap between evidence-based guidelines concerning management of ABU and clinical practice has been observed throughout the world [15,16]. Overtreatment of ABU is a quality, safety, and cost issue, particularly as unnecessary antibiotics lead to emergence of resistant pathogens [14,17].

Audit and feedback, or providing healthcare professionals with timely data about their performance, has proven efficacy as a means to improve quality of care [18,19]. Two systematic reviews of audit and feedback concluded that there was no evidence that multifaceted interventions worked better than did audit and feedback alone [18,19]. These reviews also suggest that the structure of the intervention should be tailored to the local setting and that the intensity of feedback should be high. More specific information about elements of effective audit and feedback emerges from a qualitative survey of the Department of Veterans Affairs (VA) facilities with either high or low adherence to six clinical practice guidelines [20]. VA facilities with a high level of guideline compliance provided feedback that was frequent/timely, individualized, nonpunitive, and in some cases, customizable. Additionally, providing the correct solution in the feedback appeared to be important [21]. We plan to build upon these findings using audit and feedback first at the individual level and later at the level of the hospital ward team to improve the implementation of ABU guidelines into routine care.

### Primary objectives and hypotheses

#### Objective 1

The first objective is to improve quality of care concerning catheter-associated ABU, in terms of clinical outcomes through implementation of an audit and feedback strategy at the intervention site. Clinical outcomes will also be monitored at the control site. Both sites are tertiary care VA hospitals. We hypothesize that improving adherence to evidence-based guidelines concerning ABU will decrease the inappropriate use of antibiotics to treat catheter-associated ABU (objective 1a) and will decrease inappropriate screening for ABU (objective 1b).

#### Objective 2

Our second objective is to measure increases in clinicians' knowledge of and attitudes towards practice guidelines associated with the intervention. Domains of knowledge measured will include awareness of and familiarity with ABU guidelines. Domains of attitudes measured will include acceptance of and outcome expectancy regarding nontreatment of ABU. We hypothesize that successful implementation of the intervention will improve clinicians' knowledge of and attitudes towards the guidelines.

## Methods

### Study design

The study uses a controlled pre/post design to test an intervention using audit and feedback of healthcare providers to improve their compliance with ABU guidelines. The intervention and the control sites are two different VA hospitals.

### Conceptual framework

**Figure 1:**
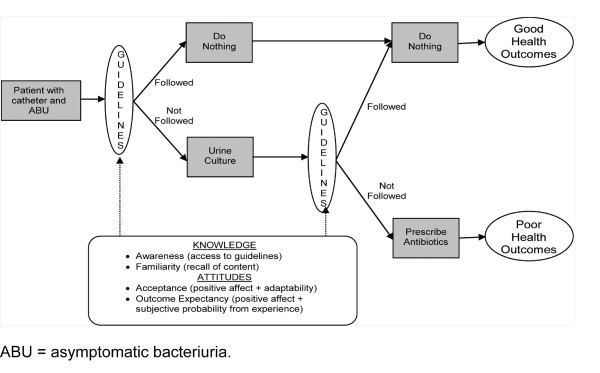
**conceptual model for treatment of asymptomatic bacteriuria (ABU) and patient health outcomes**. Our conceptual model adapts and updates elements of the Cabana model of "Why don't physicians follow clinical practice guidelines?" to focus on the following barriers to guideline implementation: awareness and familiarity (knowledge), agreement and outcome expectancy (attitudes), and external barriers (behavior). The audit-feedback intervention will tackle 2 points in the chain of events that leads from a patient at risk to a patient who receives unnecessary antibiotics for ABU: the decision to order a urine culture (inappropriate screening) and the decision to treat a positive urine culture (inappropriate prescribing). We can measure the clinical outcomes after each of these points (objective 1), and at the same time we can measure changes in providers' awareness, familiarity, acceptance, and outcomes expectancy of ABU guidelines (objective 2). This conceptual model will help us determine which aspects of our implementation protocol are responsible for the observed changes in clinical outcomes.

The conceptual framework for our study (Figure 1) has been adapted and updated from the Cabana *et al. *[22] model of barriers to physician guideline adherence to focus on the following issues: awareness and familiarity (knowledge), acceptance and outcome expectancy (attitudes), and external barriers [22,23]. The first external barrier that we will address is that the various definitions of CAUTI and catheter-associated ABU are obscure, conflicting, and difficult to apply to hospitalized patients. The ABU and CAUTI guidelines lack specific information about how to apply the definitions of ABU and CAUTI to individual patients. We will provide this clarity by developing a diagnostic algorithm to distinguish between CAUTI and ABU.

Distributing this guidelines-based algorithm will address both awareness and familiarity, but guideline dissemination alone is not an effective method to achieve guideline implementation [18,19,24-26]. The audit and feedback intervention will tackle two points in the chain of events that leads from a patient at risk to a patient who receives unnecessary antibiotics for ABU: the decision to order a urine culture (inappropriate screening) and the decision to treat a positive urine culture (inappropriate prescribing). We can measure the clinical outcomes after each of these points (objective 1); at the same time, we can measure changes in providers' knowledge (awareness and familiarity) and attitudes (social norms, acceptance, risk perceptions, self-efficacy, and outcome expectancy of ABU guidelines) (objective 2). The algorithm itself and the instructions on how to use it (via audit and feedback) will address self-efficacy concerning the guidelines. Lack of outcome efficacy will also be addressed through the audit and feedback sessions, as clinicians will be reassured when they see that withholding inappropriate antibiotics does not lead to harm and may even benefit their patients. We expect agreement with and acceptance of guidelines to likewise increase. We will also identify external barriers to guideline compliance by performing exit interviews with clinicians who have participated in the study. The conceptual model depicted in Figure 1 will enable us to determine which aspects of our implementation protocol are responsible for the observed changes in clinical outcomes.

### Study timeline

Our study will occur over three years (see Table 1). Year 1 will be the development phase, year 2 will involve the implementation of the intervention and the initial pre/post evaluation, and year 3 will provide follow-up and evaluation of the sustainability of the intervention. Throughout the three years of the study, we will perform surveillance at both sites for the clinical outcomes that are the focus of objective 1. Specific hospital units with low levels of guideline-concordant behavior and high clinical need for the intervention will be the focus of our strategy. Also during the first year we will develop the educational study materials and surveys that will be used during the second and third years of the study. These study materials include the CAUTI diagnostic algorithm, the audit and feedback intervention and script, and the surveys to be administered before and after the intervention.

**Table 1:** overview of study activities at intervention and control sites

Year	MEDVAMC (intervention)	STVHCS (control)
Year 1	Baseline surveillance	Baseline surveillance
	Development of study materials (algorithm, surveys, and audit and feedback script)	
	Qualitative data collection on study materials	

Year 2	Ongoing surveillance	Ongoing surveillance
	Guidelines distribution (algorithm)	Guidelines distribution (algorithm)
	Intervention: individual audit and feedback	
	Pre/post surveys	

Year 3	Ongoing surveillance	Ongoing surveillance
	Guidelines distribution (algorithm)	Guidelines distribution (algorithm)
	Intervention: unit-level feedback	
	Pre/post surveys	Pre/post surveys

The intervention will begin in year 2. We will distribute guidelines and definitions concerning ABU and CAUTI at both sites in the form of a diagnostic algorithm. Guideline distribution to providers will continue at appropriate intervals at both sites for the ensuing two years. During the second year, we will provide individualized audit and feedback at the intervention site. The research team will review episodes of bacteriuria and the management implemented by the provider who ordered the urine culture. Research personnel will then visit the provider to discuss whether his or her behavior was or was not in compliance with ABU guidelines. Unit-level feedback will also be given by the research assistant on a monthly basis by presenting the unit's results in graphical form. The unit in this case will be the five hospital wards for the long-term care patients and the eight internal medicine teams for the medicine wards. At the control site, algorithm distribution alone will occur during the second year. During the second year, we will also evaluate the effect of the intervention on the barriers to guidelines implementation in terms of awareness, familiarity, acceptance, and outcome expectancy through pre/post surveys completed by healthcare providers at the intervention site. Exit (qualitative) interviews with study participants will also address potential barriers to implementation of our intervention protocol.

During the third year of the study, the individualized visits to providers will cease, but unit-level feedback will continue over the course of the third year at the intervention site. During this year, the surveys will be administered at the control site in addition to the intervention site so that we can assess the effect that the surveys alone have on prescribing behavior and on responses to survey questions. The purpose of the third year is to continue to measure elements of the study, as well as to evaluate issues of sustainability and the minimal intervention elements needed for dissemination.

### Setting and participants

#### Setting

The intervention site and the control sites were chosen because they are alike in terms of ward organization, patient population, infection-control software, and clinician and medical resident involvement in patient care. We also studied the organizational context of the proposed intervention site (MEDVAMC) and control site (STVHCS) using the results of the VA Clinical Practice Organizational Survey (CPOS), Chief of Staff Module, with specific focus on responses to survey elements concerning support for guideline adherence and implementation of quality improvement measures [27]. Overall, the two sites are similar in several key elements relevant to our project. In particular, audit and feedback is not used extensively at either site and is primarily applied to clinicians' laboratory test ordering. Both sites rely on designated site champions to implement clinical guidelines or performance measures, although finding someone willing to take on this role was difficult at one of these sites because of time constraints. The leaders at both sites are strongly committed to continual improvement.

We believe that a hospital-wide intervention will be necessary (as opposed to intervening at the ward level) because medical residents travel throughout the hospital via consult services and thus, may potentially serve to diffuse knowledge throughout the facility. Hospital units at the intervention site with the highest rates of guideline-noncompliant behavior were identified, including the five general medicine wards and the five extended-care units, where 36.1% of patients received inappropriate treatment. The corresponding units at the control site have been selected for our study (three medicine wards and two extended-care wards). The intervention and control facilities are in different Veterans Integrated Service Networks, or VISNs, which is advantageous for study purposes. For the STVHCS to be a true control, healthcare providers should not overlap or have significant work-related communication with the intervention facility. Also, performing the study in two different VISNs will ultimately facilitate diffusion of the study intervention.

### Participants

#### Research team

The research team brings together a diverse group of members from both sites. The nine investigators include three infectious diseases physicians, a geriatrician, a biostatistician, a measurement psychologist, a health economist, and two industrial/organizational psychologists. Each site has a research assistant, with oversight provided by the overall research coordinator for the study. A programmer works with the team to code the database, and the research site has dedicated personnel for research-compliance assurance.

#### Healthcare providers targeted in the intervention

The audit and feedback intervention will be applied to the healthcare providers who make the decision to treat CAUTI. The healthcare provider who makes the decision to order a urine culture and to prescribe antibiotics for a positive urine culture differs depending on hospital unit. In the internal-medicine wards, this decision is made by internal-medicine residents, who are usually either interns or second-year residents (postgraduate year 1 or 2). Approximately 170 medical residents rotate through the intervention site medicine wards per year. The extended-care units are staffed by nurse practitioners and physician assistants, with supervision provided by VA staff physicians trained in geriatrics. Therefore, in the extended-care ward, the intervention will target the nurse practitioners, physician assistants, and staff physicians, currently 17 individuals.

#### Patients participating in the study

All patients in the targeted study wards at the two sites are indirectly participating in the study through our surveillance for relevant clinical outcomes. Year 1 of the study began in July 2010, so we are currently in the baseline surveillance phase. Reviews of the medical records are performed for all patients in the study wards at both sites five days per week; weekend surveillance results are captured backwards from Monday's findings. This review is performed using the VA electronic medical record, the Clinical Patient Record System (CPRS). Information collected includes bed-occupancy days, presence of any type of urinary catheter (indwelling or Foley, external or condom, intermittent, and suprapubic), whether a urine culture was sent, and the results of any urine cultures sent. Bedside visits are made in one ward per month to verify the catheter presence and type reported in the electronic medical record. Additional information is collected for each episode of bacteriuria (≥10^3 ^organisms/mL of urine). For each episode of bacteriuria, we collect information about patient demographics, the presence/absence of relevant symptoms and comorbidities, and type/duration of antibiotics given. We use this information to classify episodes of bacteriuria as ABU or CAUTI and to determine whether the provider's response to the urine culture results was appropriate or inappropriate. Surveillance data are stored in Excel (Microsoft Corporation, Seattle, WA, USA), while the individual episodes of bacteriuria are detailed in an Access database (Microsoft Corporation, Seattle, WA, USA), which also uses a computer query to verify our case classification. We use Theradoc^® ^(Hospira, Inc., Salt Lake City, UT, USA), a proprietary infection-control software package used in several VA hospitals, to extract the information about antibiotic usage from CPRS.

### Intervention

#### Development of intervention components

As the study is currently in year 1, we are developing, piloting, and validating the following study materials to be used in the intervention: a diagnostic algorithm for CAUTI versus ABU, the audit and feedback script and intervention, and the pre/post surveys (described in Data collection below) of guidelines knowledge and attitudes (see Tables 1 and 2).

**Table 2:** outcome measures for objective 2

Outcome	Measurement strategy	Measure of meaningful change
Guidelines awareness	Survey questions KA1-KA2^a^	Raw number and proportion of respondents changing from NO to YES

Guidelines familiarity	Survey question KF1 ^a^	Average increase from "do not recall" or "minimal recall" to "working familiarity" or "complete recall"

Guidelines familiarity	Survey questions KF2-KF8	Raw number and proportion of respondents changing from SD or D to SA or A

Guidelines familiarity	Survey question KF9	Raw number and proportion of respondents changing from incorrect to correct answers

Guidelines acceptance	Survey questions AA1-AA2 ^a^	Raw number and proportion of respondents changing from SD or D to SA or A

Outcomes expectation	Case scenarios OE1-OE6	Raw number and proportion of respondents changing from incorrect to correct answers

Implementation performance measures	Qualitative exit interviews	≥75% positive statements
	Capture of episodes of bacteriuria	≥95% capture of episodes of bacteriuria
	Delivery of audit and feedback	≥80% delivery to correct provider

^a^KA measures knowledge awareness, KF measures knowledge familiarity, AA measures awareness acceptance, and OE measures outcome expectancy.

A = agree; D = disagree; SA = strongly agree; SD = strongly disagree.

#### Diagnostic algorithm

The purpose of the diagnostic algorithm is to operationalize the 53-page CAUTI guidelines and the 11-page ABU guidelines so that healthcare providers can determine how to apply these guidelines to their patients. This algorithm will also be used by research personnel at both sites to classify episodes of bacteriuria as CAUTI or ABU and to determine whether use of antibiotics for the bacteriuria was appropriate or inappropriate. This algorithm has been reviewed and modified in accordance with the comments of 8 of 11 experts on the guidelines panel to establish evidence of content validity and is currently being assessed by cognitive interviewing with the target population of healthcare providers to establish evidence of construct validity. Further validation efforts will include testing convergent and predictive validity.

#### Audit and feedback intervention

The audit and feedback intervention design is closely linked to the diagnostic algorithm. We are preparing a script and a visual aid based upon the diagnostic algorithm and the various decision points in this algorithm. Individualized audit and feedback will be provided to medical residents during their ward team's designated educational time and to healthcare providers in the extended-care line during charting time. Prior to an audit and feedback visit, the research personnel will prepare a color-coded version of the algorithm that indicates where appropriate actions were taken for the episode of bacteriuria (green), where decisions were inappropriate (red), and which decision path would have been guidelines compliant (blue). An individualized audit and feedback script will be generated that covers only the decision nodes relevant to the case. This script will be presented both in PowerPoint (Microsoft Corporation, Seattle, WA, USA) and on paper for discussion. The paper copy of the color-coded algorithm will be given to the healthcare provider to review, while the contents of the script are delivered verbally by the research assistant. The information content and delivery have been carefully designed to be supportive rather than punitive, and to provide the correct answer at each decision node. The delivery of the audit and feedback script and the color-coded algorithm will be pilot tested with residents who will graduate prior to the start of the intervention and with nonphysician healthcare providers on nonstudy wards.

Ward- and/or team-level feedback will be prepared and delivered on a monthly basis in the form of colorful pie charts depicting the percentage of bacteriuria treatment decisions that were or were not guidelines compliant and how each team's performance compares to that of other teams. Our study logo will appear on all study materials to facilitate awareness and recognition of our campaign.

#### Outcomes

Objective 1 focuses on the clinical outcomes of inappropriate screening for and treatment of catheter-associated ABU, as well as on complications of inappropriately prescribed antibiotics (Table 3). Objective 2 focuses on measuring changes in knowledge and attitudes concerning the ABU and CAUTI guidelines (see Table 2).

**Table 3:** clinical outcomes for objective 1 in order of importance

Outcomes	**Measurement of change**^**a**^
Inappropriate treatment of CAABU	Fewer episodes of CAABU treated inappropriately

Inappropriate collection of urine cultures from patients with CAABU	Decreased number of urine cultures/1,000 catheter-days

Number of days antibiotics given for CAABU	Fewer days of antibiotic use for CAABU

Use of urinary catheters	Decreased urinary catheter-days/patient bed-days

Complications of inappropriate antibiotics	*Clostridium difficile *colitis, emergence of resistant organisms^b^

Complications of bacteriuria	No increase in pyelonephritis or urosepsis

^a^Comparisons are for the specific wards on the intervention facility and the corresponding wards on the control facility. ^b^In 30 days following inappropriate antibiotic use in a given patient.

CAABU = catheter-associated asymptomatic bacteriuria.

### Data collection

#### Surveillance of target hospital wards

Surveillance of all targeted hospital wards will include regular, systematic chart reviews of medical and nursing notes to identify each patient with a urinary catheter, each urine culture ordered on those patients, and the ordering of antibiotics after urine culture results are reported. Research personnel have been trained to follow a systematic process of documentation for each of these measures (described in Table 3), which has been validated against bedside observation.

#### Surveys

The survey instrument was created through literature review, review of existing antimicrobial stewardship projects, and discussion with investigators conducting related studies, including studies of UTI and ABU [28-33]. The survey contains items that measure knowledge of the guidelines and their contents, as well as items that measure attitudes and outcome expectancies related to the guidelines. No standardized instrument is available to assess physician-related barriers to appropriate management of ABU. Our starting point was prior surveys of management of hypertension [31] and antibiotic-prescribing decisions for pneumonia [29]. We also found several qualitative studies concerning prevention of CAUTI [34,35] and inappropriate use of urinary catheters [36]. Two projects addressing unnecessary antibiotic use were also relevant: the "Do Bugs Need Drugs" project in Canada [37] and the postprescription antimicrobial review study mentioned in our Background section above [38]. The principal investigator contacted the authors of these studies by email, by telephone, and/or in person at national meetings to discuss appropriate survey design.

The survey instrument will have three sections. The first section will be a cover letter. The cover letter explains the purpose of the study to the healthcare providers and explains that their participation is entirely voluntary, thus satisfying implied consent for participation. The second part of the survey is a cover sheet that assesses provider demographics, such as level of training, type of training, etc. This cover sheet will have a randomly generated number on it that will also appear on each subsequent sheet of the survey. The cover sheet with the provider's identifiable information can then be separated from the surveys to avoid bias in interpretation of survey results. The third section of the survey contains the survey questions. These questions will assess knowledge: degree of awareness of the existence of the guidelines, familiarity with the guidelines' content, and confidence in that familiarity. Other questions will measure physicians' acceptance of the guidelines, and we have also designed short patient scenarios to assess outcome expectancy about the guidelines. Each case will present a hospitalized patient with bacteriuria and a chronic, indwelling urinary catheter. The cases will differ in elements, such as the level of pyuria, the patient's age and comorbidities, the type of organism isolated from the urine, the appearance or smell of the urine, the presence of specific urinary symptoms, and the presence of vague systemic complaints. Providers will be asked whether they would or would not treat each case with antibiotics. These cases are designed to address providers' beliefs about the consequences of not treating bacteriuria (risk perception) and to elucidate which elements drive the decision to treat/not treat bacteriuria.

Although we have built our survey on established models concerning antibiotic prescribing decisions, [29] our survey instrument has been tailored to our specific clinical issue (CAUTI) and, thus, will require pilot testing with a selection of participants from the targeted groups of healthcare providers. We plan to conduct cognitive interviews to assess clarity of wording, understandability of items, and appropriateness of response options. We will conduct preliminary analyses of responses given, response-option use frequency, and floor/ceiling effects. Results of these analyses will be used to further revise items and response scales as necessary. We also will use our pilot testing to develop questions that specifically address social norms and self-efficacy.

#### Monitoring the success of the intervention

We plan to monitor the success of the intervention as we proceed, which in itself is crucial to interpreting the outcomes and ultimately disseminating the intervention to other sites. We will administer brief exit interviews when giving the postintervention surveys in years 2 and 3 (Table 2). In these interviews, we will informally assess and address barriers to implementation that may arise during the course of the study by asking providers about their perception of the implementation efforts. Although we do not expect these information surveys to result in quantifiable data, they will help us customize our intervention to local conditions in the various hospital units.

### Analysis

#### Analysis plan and sample size for objective 1

##### Independent variables for objective 1

For the variables in objective 1, it is important to understand that the intervention is applied to the healthcare providers, but the unit of analysis for the outcomes is episodes of bacteriuria occurring in catheterized inpatients (Table 3). We will account for the correlation that may exist among patients treated by the same healthcare provider in our regression models. The independent variables include age and gender of the patient, duration of catheter use, catheter type, hospital ward and service, types and quantities of organisms found in the urine, number of white blood cells (WBCs) in urine, number of WBCs in the serum, and highest temperature in 24 hours around the time of urine specimen collection.

##### Dependent variables for objective 1a--inappropriate treatment

The main outcome of interest for this objective is the number of cases of ABU that are managed inappropriately out of all episodes of bacteriuria. This determination will be made by applying the diagnostic algorithm. The dependent variable to be used in the analytic models will be whether or not each episode of bacteriuria is managed inappropriately. The unit for analysis will be episodes of bacteriuria.

A secondary outcome will be the number of days that antibiotics are given to treat ABU. We will determine the reason for treatment of bacteriuria (if stated in the medical record), antibiotics given, and duration of treatment. As a safety issue, we will monitor outcomes of bacteriuria at both sites for 30 days following the episode. These outcomes will include any side effects of the antibiotics given, isolation of an organism resistant to the antibiotics given, or *Clostridium difficile *colitis. We will also monitor for any complications that may develop from bacteriuria, such as symptomatic UTI, pyelonephritis, and urosepsis.

##### Dependent variables for objective 1b--inappropriate screening

For this objective, we are interested in whether the intervention results in decreased screening for ABU in catheterized patients. Screening for catheter-associated ABU will be measured by the number of urine cultures collected per days of catheter use. We predict that as healthcare providers become more comfortable with leaving catheter-associated bacteriuria untreated, they will recognize that urine cultures are frequently unnecessary in catheterized patients and thus, order fewer urine cultures. All urine cultures sent to the microbiology laboratory from the study units will be documented, whether positive or negative. We will calculate the number of urine cultures collected per 100 catheter device-days for each unit.

A secondary outcome for objective 1b will be the frequency of use of urinary catheters in hospitalized patients. We will need to track this information, as our intervention may have the unintentional but beneficial effect of causing providers to remove unnecessary urinary catheters. Therefore, we will also calculate the number of catheter device-days per 100 patient bed-days on each unit.

##### Estimated sample size for objective 1a--inappropriate treatment

We have estimated the sample size of episodes of bacteriuria needed at each of the two sites based on testing the differences in two independent proportions. The effect size to be detected is *h *= | φ_1 _- φ_2 _|, where φ = 2 arcsine √P [39]. Preliminary analyses found that the percentage of patients who received inappropriate treatment was 36%. Assuming a two-sided test, α = .05, and power of 80%, a sample size of 300 episodes is needed at each site in order to detect an 11% reduction in inappropriate treatment at the intervention site (down to 25%) compared to the control site rate of 36%. Specifically, *p*_1 _= .36, φ_1 _= 1.287, *p*_2 _= .25, φ_2 _= 1.047, and *h *= .24, which represent a small effect size. On the specific hospital units targeted in this study, there were over 3,000 discharges associated with urinary catheterization from the study wards at the two sites combined in fiscal year (FY) 2008. Thus, we should have adequate power to detect differences between the intervention and control sites. For the medicine units alone, we will have enough power to detect whether there was a difference in inappropriate treatment between intervention and control sites because there were approximately 1,600 patients at the Houston site estimated to have had catheters in the non-ICU medical bed sections in FY2008 and 1,300 unique patients in San Antonio.

##### Estimated sample size for objective 1b--inappropriate screening

We do not have estimates of the percentage of catheterized patients who are screened for ABU. We have based our estimates of the sample size on detecting a reduction in the intervention group of 25% in the percentage of catheterized patients who are screened for ABU. Based on estimates of the number of catheterized patients at Houston and San Antonio in a one-year time period (1,867 and 1,512, respectively), we will have 80% power to detect a 25% reduction in percentage of patients screened, even if the percentage of screened patients is as low as 10%.

#### Analyses for objective 1

For the analysis of inappropriate treatment, we will test for differences in the proportion of inappropriately treated episodes of bacteriuria between intervention and control sites using a hierarchical regression approach. Episodes will be nested within patients and patients nested within provider. Independent variables will be those described above. A significant value for the parameter estimate for the intervention will indicate that the audit feedback group differed from the control group.

For the analysis of inappropriate screening, we will conduct a logistical regression analysis in which the dependent variable will be whether or not the catheterized patient received screening for ABU. Hierarchical models will allow nesting of patients within provider. An indicator variable for the intervention effect will be used to determine differences between the audit feedback and control groups. Independent variables will include variables to indicate the unit in which the patient was hospitalized in addition to patient-related characteristics. A similar logistical analysis will be run for the secondary outcome of whether or not the hospitalized patient had a urinary catheter. For both the primary and secondary outcomes, the number of cultures per 100 device-days and the number of device-days per 100 bed-days will be compared among the units using analysis of covariance.

For the secondary outcome of days of inappropriate antibiotic use, we will first examine the distribution of the number of days that antibiotics were given. If the distribution is not normally distributed, we will consider appropriate transformations. In addition, we may consider use of hierarchical Poisson regression models.

For the remaining dependent variables identified in Table 3, when possible, we will test for differences between the intervention and control using the hierarchical regression approaches above. For rare outcomes for which fewer than 10 total cases are expected, we may be unable to conduct comparative analyses and will instead provide descriptive analyses.

#### Analysis plan and sample size for objective 2

We expect to survey 100 providers at the MEDVAMC in year 2, 100 providers at the MEDVAMC in year 3, and 100 providers at the STVHCS in year 3, resulting in 300 paired pre/post surveys. We will calculate the raw number and proportion of providers at each site whose survey responses exhibit clinically meaningful change (provider learning), as described in Table 2. Though we recognize that this measurement process will be exploratory, we expect the results to shed light on the efficacy of the intervention from a provider perspective, triangulating with improved clinical outcomes from objective 1 and thus augmenting our overall rationale for dissemination. We also intend to explore which of the attributes measured in objective 2 are associated most strongly with changes in clinical outcomes measured in objective 1 and to assess how well any empirical relationships are supported conceptually. Finally, we will assess the empirical relationships between provider learning and provider demographics.

To measure the success of the audit and feedback system, we will document how many episodes of bacteriuria were audited (underwent medical-record review), how many audited episodes resulted in feedback (a phone call or visit to the provider), and how many episodes of feedback were delivered to the correct provider (someone directly involved in the prescribing decisions for the patient). The information about correct delivery of feedback will be particularly important, and we expect the majority of the application barriers to occur in this area. This assessment will be made every three months by the research coordinator. If fewer than 90% of episodes of bacteriuria receive audit, or if fewer than 80% of audits are delivered to the correct team within three days, we will reassess our audit and feedback methods. These evaluation elements will be critical for developing implementation and dissemination protocols.

### Ethical approval

This study protocol has been approved by the institutional review board at Baylor College of Medicine as protocol H-24180, by the Research and Development Committee at the Michael E. DeBakey Veterans Affairs Medical Center as protocol 08K06.H, and by the University of Texas Health Science Center at San Antonio as protocol HSC20100128H. This proposal was reviewed at the August 2009 meeting of the Health Services Research and Development Service (HSR&D) Scientific Merit Review Board (SMRB), and the Board funded this proposal as IIR-09-104.

## Discussion

Our intervention is tightly focused on a specific aspect of poor-quality care that has been observed in both our specific setting [15] and in other hospitals throughout the world [16]. We propose a strategy based on what has been successful in analogous guideline-implementation research [38]. If successful, our proposed intervention will significantly improve the quality of healthcare delivered to hospitalized patients with urinary catheters. Dissemination of our intervention could occur by making it a component of the bladder bundle programs currently being introduced in hospitals throughout the United States. In addition, studies evaluating the comparative and cost effectiveness of the individualized and ward-level audit and feedback are needed.

## Competing interests

The authors declare that they have no competing interests.

## Authors' contributions

BWT conceived of the study and drafted and revised the protocol with considerable input from the entire research team (ADN, SH, HK, PAK, NP, JEP). Research assistant KSL helped with the drafting of the manuscript. Graduate student HK is leading the design of the audit and feedback intervention. All authors have read and approved the final manuscript.
